# Design Considerations for a Surface Disinfection Device Using Ultraviolet-C Light-Emitting Diodes

**DOI:** 10.6028/jres.126.045

**Published:** 2022-02-16

**Authors:** Pratibha Sharma, Pao Chen, Saya Han, Peter Chung, Jungpin Chen, Justin Tseng, Chang Han

**Affiliations:** 1 Violumas/Cofan Komot USA, Vaughan, ON L4K3X2, Canada; 2 Violumas Inc., California 94538, USA; 3 Cofan USA, Taoyuan City 333, Taiwan; 4 Violumas Taiwan, Lung-tan, Tao-Yuan 32556, Taiwan

**Keywords:** device design, light-emitting diode, simulations, surface disinfection, ultraviolet-C, UV-C disinfection

## Abstract

Ultraviolet-C (UV-C) radiation, spanning wavelengths between 200 nm and 280 nm, has proven germicidal qualities and medical, industrial, and environmental applications. The need for new disinfection technologies and the prospect of eliminating mercury-based radiation sources compels research on ultraviolet (UV) light-emitting diodes (LEDs). UV-LED technology could be used for customized and point-of-use products for disinfection and sterilization. We focused on the design and development of a surface disinfection device using UV-C LEDs, including potential user targets, important design parameters, and final validation methods. Optical and thermal simulations were used to illustrate the design process and associated challenges. A sample device prototype was developed, and microbial validation results are presented.

This article was sponsored by Dianne L. Poster, Material Measurement Laboratory, and C. Cameron Miller, Physical Measurement Laboratory, National Institute of Standards and Technology (NIST). It is published in collaboration with the International Ultraviolet Association (IUVA) as a complement to the NIST Workshop on Ultraviolet Disinfection Technologies, 14–15 January 2020, Gaithersburg, MD. The views expressed represent those of the authors and not necessarily those of NIST.

## 1 Introduction

Germicidal ultraviolet-C (UV-C) radiation, with wavelengths ranging from 200 nm to 280 nm, has been proven to be effective against viruses, bacteria, and other pathogens by damaging their genetic material and obstructing pathogenic multiplication [1]. With the coronavirus disease 2019 (COVID-19) pandemic, caused by the severe acute respiratory syndrome coronavirus 2 (SARS-CoV-2), and healthcare-associated infections (HAIs) [2, 3] still presenting problems, disinfection strategies have gained importance to mitigate risk. Between 20% and 40% of HAIs occur because of cross-contamination from hands of personnel, and there is also evidence of transmission from fomites or surfaces [4]. Most viruses remain viable longer on nonporous fomites relative to porous fomites, although there are exceptions [4, 5]. Current surface disinfection strategies for fomites are mostly chemical-based methods, and, depending on the microbial targets, chemical constituents, and dilutions, these chemicals can have contact times ranging from a few seconds to up to 10 min. These methods are personnel dependent, which can potentially limit their effectiveness. With the increase in antibiotic-resistant microbes [6], HAIs pose a serious health threat and can increase hospital stays and mortality rates. While the use of chemical disinfectants is increasing [7], the need for automated, chemical-free technologies has made ultraviolet (UV) radiation a suitable alternative. More than a decade ago, it was thought UV exposure had minimal effects on viral survival in indoor environments [4]; however, recent studies have shown otherwise (see Poster *et al.* [3] and references within).

Most UV-based disinfection devices on the market use a low-pressure mercury lamp with a peak wavelength of 254 nm [8]. Mercury lamps are powerful radiation sources that can destroy microbes within seconds after irradiation. Increasing requirements in printing, curing, horticulture, sterilization, and disinfection (among others) have necessitated longer-lasting, lower-maintenance solutions. In addition, the inflexible form factors of UV lamps and their long start-up times are hurdles for implementation in some applications. The toxic mercury content makes them environmentally dangerous as well [9]. Hence, many industries, such as water and air disinfection, are replacing mercury lamps with UV light-emitting diodes (UV-LEDs) [10].

UV-LEDs are commercially available in a variety of peak wavelengths, which has led to applications not attainable with the use of mercury-based UV lamps. While surface disinfection using UV-LED technology seems straightforward, several design complexities need to be addressed. In this paper, we present design considerations for the development of a surface disinfection device using UV-C LEDs. We emphasize the design parameters that need to be understood in relation to application-specific requirements. We present details on the design and development of a surface disinfection device using high-power UV-C LEDs.

## 2 Identification of User Targets

Identification of user requirements forms the first step in the development of a device. These requirements can be gathered from information on the targeted usage, the intended market, and the performance requirements for the device. In the following sections, we look at some of the requirements that need to be considered for the development of a surface disinfection device.

### 2.1.1 Targeted Microbes and Inactivation Requirements

**Fig. 1:**
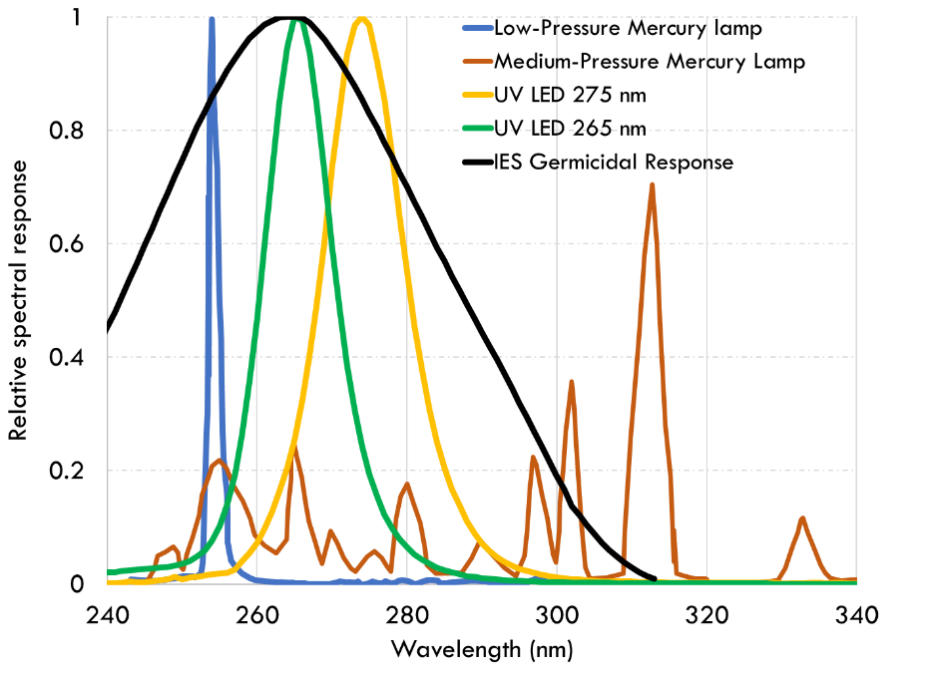
Relative spectral output as a function of wavelength for various UV light sources in relation to the Illuminating Engineering Society’s germicidal response data, adapted from Refs. [12, 13]. UV-LED relative spectral measurement data are superimposed.

Microbial inactivation targets can vary depending on the targeted application environment for the device. In the case of healthcare, inactivation levels of 4 log_10_ units (99.99% reduction of viable pathogens)^1^1 4 log_10_ units refers to a 99.99% reduction, calculated as log10 (*N*0/*N*), where *N*0 is the initial value, and *N *is the final value. or greater may be a regulatory requirement, while this may be reduced to 3 log_10_ inactivation (99.9%) for household use. This requirement may have to be substantiated, keeping in mind the international markets and the corresponding regulatory bodies. While standards and regulations have not yet been established globally, published studies and recommendations can be referenced while designing disinfection devices [11]. Spectral sensitivity may be represented by a generic germicidal effectiveness curve, based on deoxyribonucleic acid (DNA) absorption. Germicidal effectiveness is greatest between approximately 250 nm and 280 nm (Fig. 1). A light source with a peak wavelength within this range is considered to be suitable for disinfection. Figure 1 also shows the relative spectral power distribution of different light sources, including our measurements for UV-LEDs. While the medium-pressure mercury lamp shows a broadband spectral output, the low-pressure mercury lamp emits at a peak wavelength of 254 nm [12]. Commercial UV-C LEDs are typically available at peak wavelengths of 265 nm and 275 nm, with the 265 nm wavelength coinciding with the peak of the germicidal response data published by the Illuminating Engineering Society (IES) [13].

**Fig. 2:**
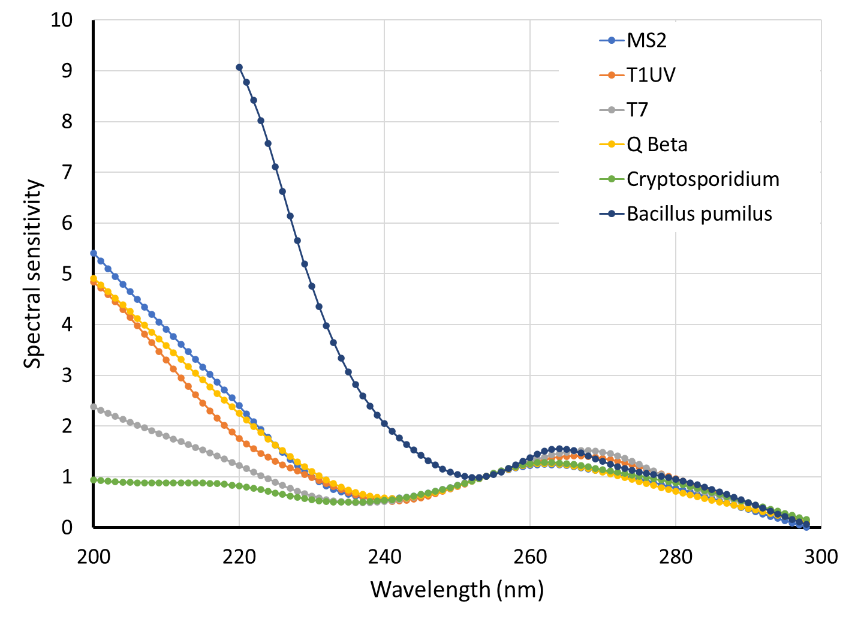
Spectral sensitivity as a function of wavelength for different microbes, drawn using data from Ref. [15].

While a generic spectral sensitivity curve may be a good starting point, microbes exhibit “action spectra,” which provide detailed information on the effectiveness of UV for their inactivation [14]. Figure 2 shows the spectral sensitivity of MS2, T1UV, T7, and Q beta bacteriophage species, along with *Bacillus pumilus* and Cryptosporidium, indicating a peak near 265 nm within the 250 nm to 280 nm band [15]. The response is significantly different at shorter wavelengths, indicating the differences in inactivation as a function of wavelength.

### 2.1.2 Exposure Times

UV dose is a function of UV irradiance and exposure time [1] as represented by Eq. (1).

$UV\;Dose\left(\frac{mJ}{{cm}^{2}}\right)\;=UV\;irradiance\;\left(\frac{mW}{{cm}^{2}}\right)\times \;exposure\;time\;(s)$ (1)

Inactivation UV doses vary depending on the microbe (Table 1). For example, Methicillin-resistant *Staphylococcus aureus* (MRSA) requires <10 mJ/cm^2^ of UV to obtain a 3 log_10_ reduction, but *Clostridioides difficile* (*C. difficile*) requires a significantly higher UV dose to achieve the same results. Recent studies have shown that SARS-CoV-2 requires a dose less than 4 mJ/cm^2^ for 99.9% inactivation [16]. Blatchley *et al*. provided a summary of dose-response behavior indicating the potential of UV-C to inactivate SARS-CoV-2 [17].

Depending on how the UV device is used within the targeted environment, exposure time requirements vary. While shorter exposure times may be more desirable, limits on achievable UV intensity may restrict this flexibility.

### 2.1.3 Objects To Be Disinfected

Knowledge of the targeted object(s) to be disinfected, including the shape, dimensions, material properties, and surface type, is important to ensure disinfection effectiveness. Since UV radiation is a line-of-sight technology, in terms of the surface disinfection application, targeted UV dosage must be ensured on the entire area that needs to be disinfected. Shadowing must be avoided, and knowledge of minimum irradiance can be used as a metric to ensure performance effectiveness. In addition, information on material properties such as transmittance, reflectance, *etc*., is required if layered media need to be disinfected.

**Table 1:** UV-C dose required for 3 log_10_ inactivation of different microbes.^a^

Microbe and Reference	Dose for 3 log_10_ (99.9%) Reduction^b^ (mJ/cm^2^)
*Acinetobacter baumannii* [18]	3.3
*Salmonella typhimurium* (LT2 SL3770) [19]	7.8
Methicillin-resistant *Staphylococcus aureus* (ATCC BAA-1556*)* [19]	8.8
*Klebsiella pneumoniae* [19]	10
*Enterococcus faecium* (Vancomycin-resistant) [19]	11
*Pseudomonas aeruginosa* (ATCC 10145) [19]	6.8
*Escherichia coli* (ATCC 29425) [19]	23
*Enterococcus* spp. [20]	37
*Clostridioides difficile* (JCM 1296; endospores) [19]	17 (far-UV-C)
SARS-CoV-2 (2019-nCoV/ItalyINMI1) [16]	3.7

^a^ A recent publication from Masjoudi *et al*. [19] showed compiled data from over 250 studies. Data obtained using UV-C LEDs have been chosen, wherever available.

^b^ The dose for *C. difficile* is for a far UV-C (222 nm) wavelength.

Porous media with complex layered structures, such as N95^2^2 “N95” is a filter class designation of the U.S. National Institute of Occupational Safety and Health (NIOSH). It is applied to respirators that are at least 95% efficient at filtering NaCl aerosols with particle sizes of mean diameter 75 nm ± 20 nm (NIOSH Procedure No. TEB-APR-STP-0059, December 13, 2019). masks, are more difficult to disinfect as compared to simple, nonporous objects such as cell phones and tablets. So, device designers must take these factors into consideration when determining UV intensities. Recent studies of N95 mask disinfection have been provided by Geldert *et al*. [21] and Chandran *et al.* [22], and the latter includes UV-LEDs.

### 2.1.4 Mechanical Limitations

For an enclosed disinfection device, the dimensions of the largest object to be disinfected must be known to determine the maximum volume of the disinfection chamber required. If multiple objects are to be disinfected simultaneously, all the additive volumes as well as their placement need to be incorporated in the mechanical design. Working distances as well as thermal management components required to achieve minimum intensity requirements would also affect the mechanical device dimensions. If the disinfection device is handheld, then there is greater flexibility in device design because the device can be moved to cover a larger area; however, human exposure to UV may be a concern. It may also imply variable performances based on working distances and user handling. In addition, there might be limitations on device size based on the usage environment. Device weight can also be a limitation, particularly for a handheld or a portable device. If the device utilizes fans for cooling the LEDs, the noise generated should also be considered.

## 3 Device Design with UV-C LEDs

The basic principle of UV-LED operation differs significantly from that of a UV mercury lamp. While LEDs are replacing lamps in many applications, LEDs cannot be used as retrofitting sources in lamp applications. Being a semiconductor device, the operation of UV-LEDs is governed by the current-voltage (I-V) curve provided by UV-LED manufacturers. The light intensity is tunable, unlike UV lamps, and it is directly proportional to input drive current, which makes the LEDs suitable for variable-intensity applications. UV-LEDs also have the advantage of narrowband spectral output. This implies that most of the energy would be emitted in the narrow band, targeted toward specific peak wavelengths specifically suited for microbial inactivation as well as spectroscopy applications. While UV-LEDs offer advantages, product designers need to be familiar with the parameters that must be evaluated for a surface disinfection device. These are described in the following subsections.

### 3.1 Peak Wavelength

Peak wavelength relates to the wavelength at which the spectral output reaches its highest intensity and is defined by epitaxial growth layers on the wafer. After the wafer is diced into chips, the chips are segregated into bins based on peak wavelength, within a tolerance parameter (typically 3 nm to 5 nm). While UV-LED emissions are narrow, the spectral output does spread into a wavelength band resembling the Gaussian function. For this reason, the bandwidth, normally expressed in full-width at half maximum (FWHM), is useful for system designers to represent the amount of radiant flux that is emitted at the peak and at nearby wavelengths (band of about 10–12 nm). Another factor to consider is the radiant efficiency, which is the ratio of the radiant flux emitted to the electrical power consumed by the LED. The peak wavelength should be chosen based on the action spectrum of the microbe, which is identified when determining user targets.

### 3.2 Electro-Optical Performance

**Fig. 3:**
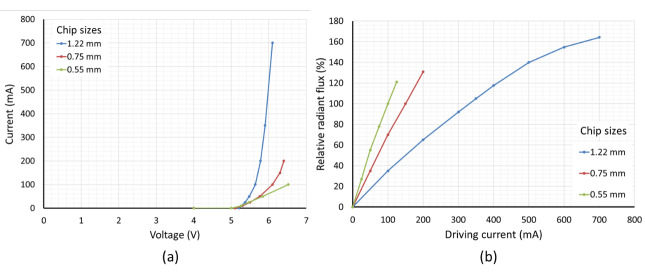
(a) Current-voltage curve for three chip sizes of a 265 nm UV-LED. (b) Relative radiant flux as a function of drive current for three chip sizes of a 265 nm UV-LED. The relative radiant flux values are taken at the typical driving current for each chip size, adapted from Ref. [23].

While the electrical characteristics and the optical output may appear to be two different parameters, their interdependency cannot be overstated, and hence we look at these in tandem. Optical output is directly affected by the drive current, and analysis of the forward current *vs*. relative radiant flux and the forward voltage *vs*. forward current graphs is essential to understand how the optical intensity varies. Figure 3 shows an example of a current-voltage curve and radiant flux as a function of drive current for three different chip sizes of a 265 nm UV-C LED (adapted from Ref. [23]). The relative radiant flux is normalized by the flux value obtained at a drive current of 350 mA for the 1.22 mm chip size, 150 mA for the 0.75 mm chip size, and 100 mA for the 0.55 mm chip size.

### 3.3 Optics

Optical output is dependent not only on the LED efficiency, but also on the type of optics and reflective materials. UV-LED packages typically emit within a 120° to 130° beam angle, and in many applications secondary optics are required to focus the light within a certain area. Beam angle refers to the angle between the two directions where the intensity is reduced to 50% of the central maximum intensity. The choice of secondary optics is important to ensure the uniform distribution of light within the targeted area. In addition, optical properties, such as the transmission within the specific wavelength range, are also important to evaluate optical losses.

Additionally, UV-LEDs may be packaged as single-chip devices or multiple-chip arrangements called LED arrays. Customized LED array designs with various chip sizes can be used to ensure targeted radiant intensities in specific locations. Hence, optimization of LED numbers and locations is essential. Uniform distribution of power density can prevent early failures and influence thermal design.

While analytical calculations can be made as a starting point for geometric arrangement considerations, optical simulations play an important role in the design process, aiding in the optimal selection of materials, determination of losses, and estimation of intensities and cost-effectiveness [24]. Setting up realistic optical models can be challenging and requires thorough knowledge of material properties (such as reflectance and transmission) as well as irradiance distributions of the source. Recent studies have compared reflective properties of materials exposed to UV-C radiation [25]. In addition, Yates *et al.* [26] have reviewed the effects of UV-C radiation exposure on aircraft cabin materials.

**Fig. 4:**
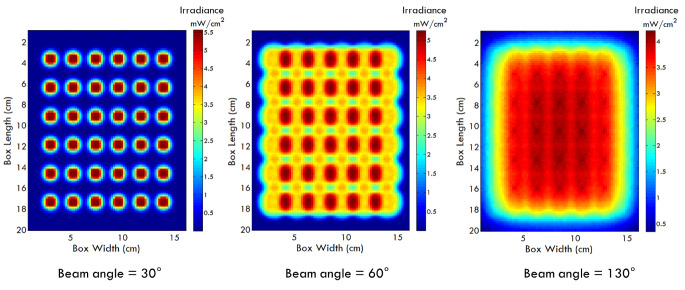
Variation in irradiance distributions with varying UV-LED beam angles. Irradiance hot spots are predominant with lower beam angles and lower working distances.

For example, Fig. 4 shows a comparison of simulated irradiance distributions obtained with different LED beam angles at a short working distance within a chamber irradiated with a UV-LED array. Minimum irradiance values as well as uniformity ratios are useable acceptance metrics for a design. It should be well understood that knowledge of the lowest irradiance values (*i.e.*, cold spot values) obtained via optical simulations is essential to determine effectiveness. The narrow beam angles provide a higher peak irradiance but a lower uniformity at this working distance. Product designers can use this information to make design choices. Simulations can also be performed in a three-dimensional space to represent complex objects, but meshing conditions need to be optimized to manage accuracy and computational times.

### 3.4 Powering the LEDs

Once the number and location of LEDs and the drive current are determined, a suitable power supply unit (PSU) or a driver must be chosen to drive the LEDs. The selection of an optimal power supply is vital to obtain the desired optical output, expected lifetimes, and reliability from the LEDs [27]. Using an incorrect power supply can both damage the LED array and be a source of dangerous hazards. Hence, the power supply should be chosen or designed keeping in mind the regulations and safety certifications as well as the specific requirements of the application.

**Fig. 5:**
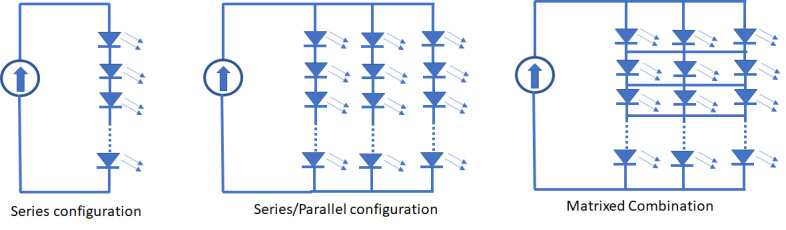
Different LED array formats define the voltage-current requirements for the PSU. LEDs may be connected in series or parallel configuration or in a matrixed combination in a chip-on-board format.

When selecting a commercially available PSU that will be connected to available power, a suitable AC to DC converting PSU should be chosen. Depending on the number of LEDs used, and the commercial availability and cost of PSUs, a circuit topology (series or parallel connections) for the LED array is needed. Figure 5 shows some examples of LED configurations.

As an example, if a UV-LED chip with a forward voltage of 6 V and a drive current of 500 mA is to be arranged in a 30 chip LED array, it can be placed in a 5× series/6× parallel configuration to work with a 30 V and 3 A PSU, or it can be arranged in a 6× series/5× parallel configuration to work with a 36 V and 2.5 A PSU.

Similar to visible LEDs, UV-LED arrays can be driven by a constant-current or a constant-voltage LED driver. A constant-current driver can be used to directly drive the array. In contrast, the constant-voltage driver requires additional circuitry to limit the current. In addition, it should be determined whether the LED array would be operated at a constant current or pulsed. Certain PSUs also allow intensity control using pulse-width modulation with resistive or DC voltage inputs, enabling the use of smart controls for integration with Internet of Things (IoT) technologies. If the LED array is to be powered using a battery, then the battery voltage and current capacity should be considered for the application.

### 3.5 Thermal Management

With lower efficiencies than their visible-light counterparts, thermal effects are even more pronounced in UV-C LEDs, from which more than 90% of the energy may be lost as heat. The reliability and lifetime of LEDs are typically judged based on depreciation of light output, which is directly related to the junction temperature. The junction temperature of an LED is not just a performance indicator of the thermal design; it also affects end-product design as the light distribution, whether it is for a water disinfection application or a surface disinfection product. Thermal management is achieved using external components such as heat sinks or fans and affects the mechanical design.

The primary thermal emission source in the LED package is the heat generation at the junction due to nonradiative recombination processes [28]. Joule heating due to the series electrical resistance of the diode and potentially at the interconnects is also another reason for heat generation. At high current levels, the contribution of parasitic resistance is significant. The relationship between the junction temperature and the current level is described by Eq. (2) [28].

$T_{J}=T_{C}+R_{th}\;(I_{f}\times V_{f}-P_{opt})$ (2)

where

*T*_j_ is the junction temperature,

*T*_C_ is the case temperature,

R_th_ is the junction-to-case thermal resistance,

*I*_f_ is the forward driving current,

*V*_f_ is the forward voltage, and

*P*_opt_ is the power dissipated optically.

The combined effects of the LED chip and the connection to the carrier form the *thermal resistance* of the package. Thermal resistance can be one of the metrics used to evaluate and control junction temperatures.

**Fig. 6:**
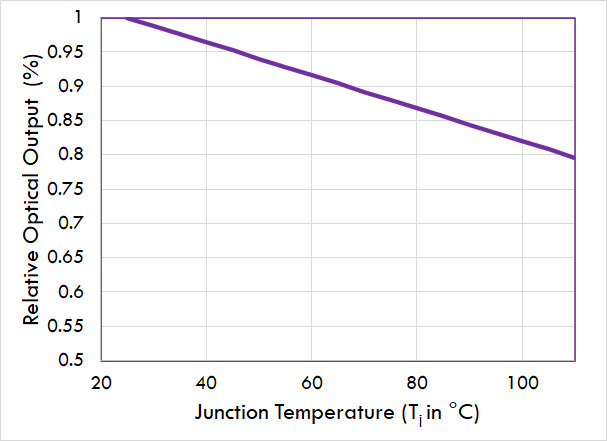
Relative optical output as a function of junction temperature at a fixed driving current for a UV-C LED.
• Efficiency: UV-LED efficiencies decrease with an increase in the junction temperature due to the reduction in optical output for the same driving currents.
• Reliability and lifetime: LED lifetimes are directly affected by junction temperatures, with reduced junction temperatures known to increase lifetimes. A 10 °C to 15 °C increase in *T*_j_ reduces the lifetime by half [28].

High junction temperatures affect the following:

• LED material quality: An increase in junction temperatures leads to temporary or permanent degradation in the different layers of the LED and affects reliability and lifetime.
• Light output: Nonoptimal thermal management causes LED junction temperatures to increase. For example, the optical output is reduced at higher junction temperatures (Fig. 6). At higher drive currents, the optical output drops even further.

In summary, optimal thermal management is essential to ensure device lifetimes and optical output. Advanced thermal management techniques at both the package and system levels help to reduce thermal resistance and junction temperature.

#### 3.5.1 Package Level

Different packaging technologies are available for commercial UV-C LEDs with varying thermal resistance. This is the first level at which interventions can be made to improve thermal performance.

**Fig. 7:**
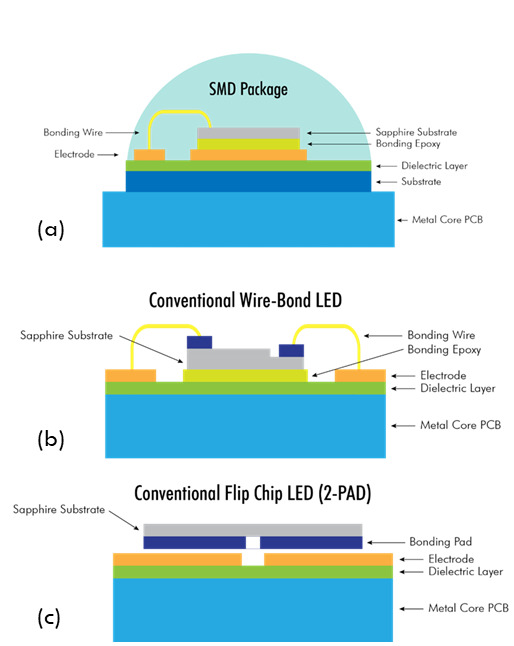
Different packaging technologies used in manufacturing commercial UV-LEDs: (a) surface mount devices (SMDs), (b) conventional wire-bond technology, and (c) standard flip-chip technology (two-pad technology). PCB is printed circuit board.

Surface mount devices are popular at the prototyping stage, providing engineers much flexibility in device design. Figure 7(a) shows the various layers that contribute to the thermal resistance of such devices, starting from the die substrate to the die attach and to the metal core printed circuit board (MCPCB) dielectric. This package typically offers high thermal resistance of about 6 °C/W or more.

In a wire-bond chip-on-board (COB) solution, a lateral LED chip is bonded on the MCPCB substrate by bonding epoxy and connects to the circuit electrodes via two bonding wires as shown in Fig. 7(b). The heat generated by the LED chip is dissipated through the chip’s sapphire substrate and bonding epoxy, followed by the dielectric layer of the MCPCB, before reaching the metal core, which typically connects to an external heat sink. This kind of packaging offers a thermal resistance of about 3 °C/W.

In a conventional flip-chip COB or two-pad COB, the LED chip is directly bonded on the circuit electrodes without the bonding wire and epoxy. The thermal dissipating path excludes the sapphire substrate and bonding epoxy, which are materials that possess higher thermal resistance. The heat is led through the chip bonding pads, circuit electrodes, and MCPCB dielectric layer and then diffused into the metal core as depicted in Fig. 7(c). In this packaging type, the thermal resistance drops to about a third of that of the wire-bond COB.

**Fig. 8:**
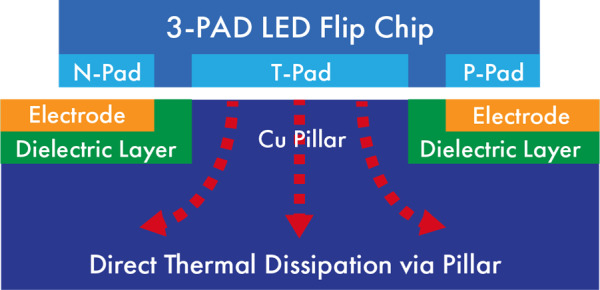
Structure of a three-pad UV-LED with a thermal pad and a copper pillar that direct the heat away from the LED.

In order to further reduce the thermal resistance, techniques such as addition of a third thermal contact pad can be applied. A three-pad flip-chip LED consists of a third contact pad, known as the thermal pad, that is electrically isolated and positioned between the *n-* and *p-*electrode pads. The thermal pad is designed as a thermal coupling window to optimize the thermal dissipation from the LED’s junction to MCPCB’s super pillar structure, which appears as an extension of the metal core inside the MCPCB. Figure 8 shows an example of such a three-pad package, with a Cu pillar, where the entire thermal dissipation path is composed of high-thermal-conductivity materials. The thermal resistance between the LED junction and COB bottom is, hence, further reduced to minimize the thermal decay. This technology allows for a 0.2 °C/W thermal resistance, which is significantly lower than that of the traditional technologies. It enables a lower junction temperature, a higher optical output, and a much longer lifetime as compared to the conventional flip-chip technology.

**Fig. 9:**
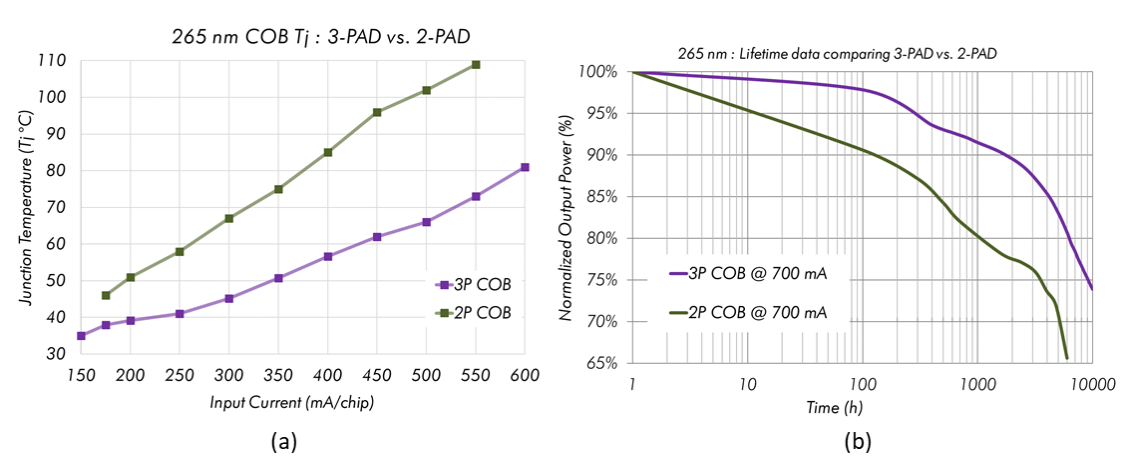
Experimental results comparing the two-pad and the three-pad technology, showing (a) the junction temperature as a function of driving current and (b) the normalized optical output as it degrades with time.

Advanced thermal management techniques such as addition of a third thermal pad can be used to drive LEDs at a higher current while maintaining lower junction temperatures and accomplishing lower thermal budgets. Figure 9 shows experimental results comparing the junction temperature as well as the lifetimes of standard two-pad and the example three-pad COB operated at the same driving current.

As shown in Fig. 9, the advantages of the thermal pad (three-pad) technology are more pronounced at higher drive currents, and higher-wattage modules are attainable without a loss in optical output. A junction temperature drop of more than 30 °C is possible depending on drive currents. In terms of the lifetime, an increase of more than 4000 h can be observed with the three-pad technology, making UV-LED adoption more feasible. Some implementations of this technology have used metal-lined holes or thermal vias underneath LED thermal pads [29], or they have utilized thermally conductive dielectric material [30].

#### 3.5.2 System Level

**Fig. 10:**
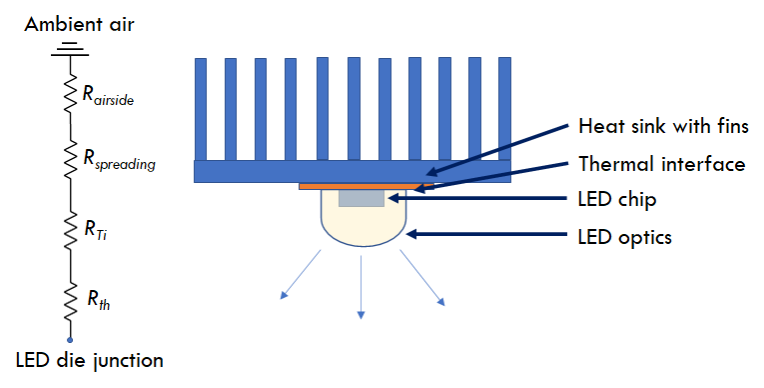
Heat-exchange path in an air-cooled heat sink system, adapted from Ref. [28].

Thermal management at a system level may be implemented via passive or active heat transfer mechanisms. In UV-LED systems using passive cooling, a metal heat sink may be used to dissipate the heat and transfer it to the surrounding air. Figure 10 shows the heat-transfer path in such a system [28], where a surface with a larger area is used to dissipate the heat from a smaller-footprint LED. While the junction-to-case thermal resistance is package dependent, the resistance from the thermal interface (*R*_Ti_) can be reduced significantly by optimal thermal material selection. The next step is the transfer of heat from the thermal interface to the larger heat sink. The effectiveness of this system can be increased by increasing the area of the extended surfaces, for example, by increasing the number of fins or by making the fins longer, or by changing the heat sink material. However, such design choices may imply a heavier and a bulkier device. Finite element analysis and computational fluid dynamics tools can help in optimizing such designs without incurring additional costs.

Such systems use natural convection as the primary means of heat removal from the system and are ubiquitous in most UV-LED–based systems due to their long lifetimes, low to no maintenance costs, and lack of power consumption. Forced convection using fans or air jets is frequently used as well to reduce system sizes and improve performance [28].

In addition, for high-wattage UV-LED arrays, fluid cooling methods such as microjet cooling [31] and microchannel cooling [32], which have been previously utilized for visible LEDs, may be used. Use of thermo-electric coolers [33] can also be considered based on application requirements and budget constraints.

### 3.6 Device Validation

Prototype device validation is extremely important to ensure that user targets are reached. There can be several ways of validating device functioning, but in terms of ensuring UV dose and disinfection effectiveness, the following two methods may be adopted.

• Radiometric measurements: Measurements using a spectroradiometer suitable for the required wavelength range can provide UV intensity values that can be compared with the simulated values. Such measurements should be performed over the entire disinfection area and at various working distances or for worst-case scenarios depending on the shape and size of the targeted disinfection objects.
• Microbiological validation: To validate the effectiveness of the device, microbial testing must be conducted using predefined protocols. In many cases, the target microbe is unavailable, and so surrogate microbes are used. Typically, standard approved practice requires testing with three replicates and controls with and without the UV light. Surrogates for different media may also be used instead of the actual disinfection objects. For example, a painted glass slide may suffice as a surrogate for a flat, nonporous medium such as a cell phone or a tablet, and testing may be performed at different UV doses to obtain the log reduction trends.

Photochromatic ink indicator cards/dosimeters are also another inexpensive means to verify UV-C dose [34], specifically in the field. However, wavelength dependence and the effect of ambient temperatures should be considered when using such methods for validation.

## 4 Model Disinfection Chamber

**Fig. 11:**
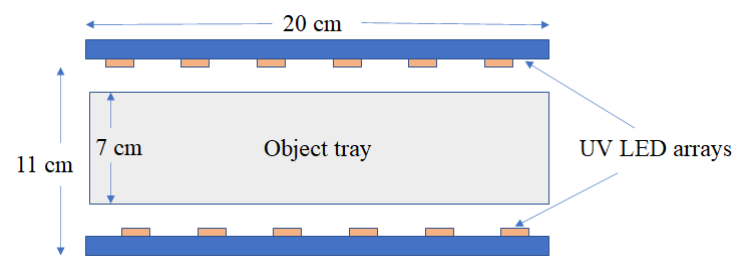
Cross section and dimensions of the UV-LED chamber.

We present the design, modeling, and validation results of a UV-LED disinfection chamber built using the three-pad UV-C LEDs. The chamber was built using a stainless-steel body to disinfect nonporous personal items such as cell phones and targeted to provide >3 log_10_ reduction of microbes such as MRSA, *E. coli*, and SARS-CoV-2. A cross section with chamber dimensions is shown in Fig. 11.

### 4.1 Opto-Electrical Design and Simulations

**Fig. 12:**
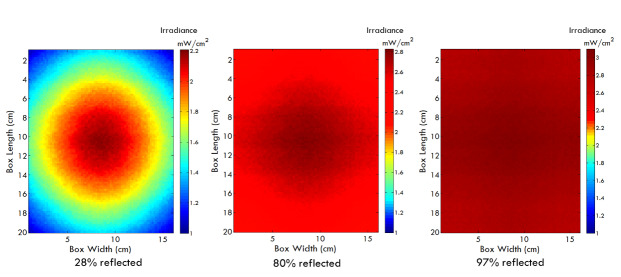
Variation in irradiance profiles with varying degree of side-wall reflections.

Standard, commercially available software (LightTools and Zemax)^3^3 Certain commercial equipment, instruments, or materials are identified in this article to specify the experimental procedure adequately. Such identification does not imply recommendation or endorsement by the National Institute of Standards and Technology, nor does it imply that the materials or equipment identified are necessarily the best available for the purpose. was used for optical simulations to determine the optimal number and location of LEDs. The chamber consisted of custom COB LEDs designed and manufactured for this prototype device, ensuring a minimum of 2.5 mW/cm^2^ optical intensity on all surfaces at a working distance of 9 cm from the COB. Each LED COB consisted of 36 pieces of 265 nm UV-C LEDs, placed at the top as well as the bottom of the chamber. The beam angle was chosen to be 130° for our design. The LEDs were operated at a driving current of 700 mA/chip. Reflective materials can have a significant effect on the uniformity and dosage of light within the chamber [35]. The effect of different types of reflective materials on the side walls of the chamber was simulated. Figure 12 shows the simulation results for three different types of reflective materials at a working distance of 9 cm from the LED array.

The overall intensity and uniformity increased when a highly reflective material was used for the side-walls of the chamber (Fig. 12). However, material costs can be a trade-off, with costs varying from $50/m^2^ to $500/m^2^, and so we chose an aluminum sheet with 80% reflectance for our prototype device.

The power supply chosen was a commercially available Underwriters Laboratories (UL) approved power supply suitable for constant-current operation of the LED array. Since the arrangement was optimized to be a 6 series/6 parallel arrangement, the PSU was chosen based on the forward voltage and the current required to drive this circuit.

### 4.2 Thermal Design and Simulations

**Fig. 13:**
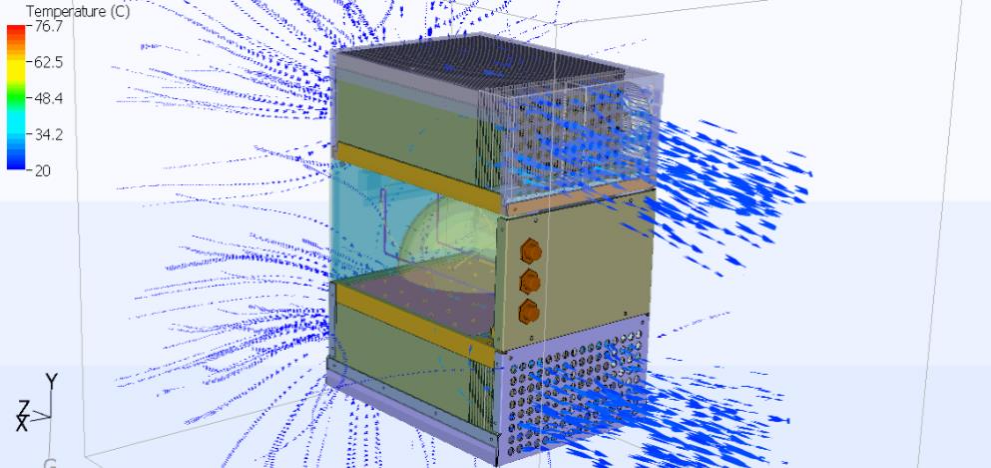
Thermal simulations performed with heat sinks and fans for the surface disinfection device.

We simulated a worst-case thermal scenario in our thermal simulations using 6SigmaET by including an individual lens and a metal heat sink and fans to dissipate the heat to the ambient air. Figure 13 shows a static image of our thermal simulations based on the prototype device.

We also conducted comparative studies with a corresponding two-pad arrangement assuming the same number of LEDs and the same drive current. With these comparable settings, we could see a maximum junction temperature difference of about 30 °C between the two-pad and the three-pad technologies, as shown in Table 2. We could potentially improve the junction temperature with the two-pad technology by about 10 °C by either changing the heat-sink material or by increasing the number of fins on the heat sink or improving the fan performance or a combination of these. However, this would lead to an increase in the thermal budget, which would in turn affect the mechanical design, making the unit bulkier. Essentially, the integrated three-pad technology and its reduced junction temperature enable high-wattage UV-LED systems to be built with nominal thermal budgets and increased lifetimes.

**Table 2:** Maximum junction temperatures obtained using thermal simulations with the two packaging technologies.

**Thermal Management Solution**	**Maximum Junction Temperature (°C)**
Three pads, Al fins, standard fan	65.3
Two pads, Al fins, standard fan	95.1
Two pads, Al fins, high revolutions per minute (RPM) fan	91.3
Two pads, Cu fins, high RPM fan	89.5
Two pads, additional Cu fins, high RPM fan (expanded heat sink)	88.3

### 4.3 Device Validation

In order to ensure performance effectiveness, device validation was carried out using in-house optical intensity measurements and microbial testing at a third-party laboratory.

#### 4.3.1 Irradiance Measurements

**Fig. 14:**
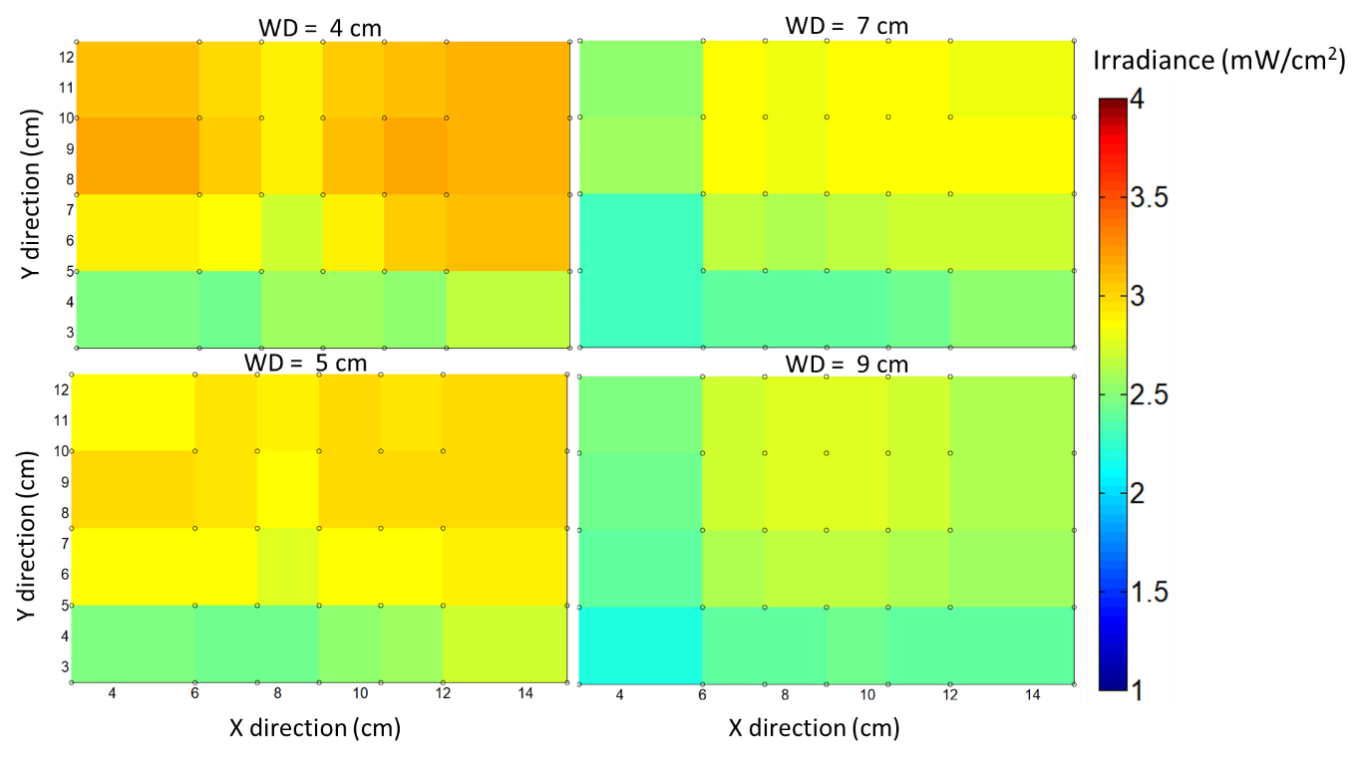
UV irradiance data measured at different working distances with only the top UV-LED array turned ON.

Irradiance measurements were made at different working distances from the top UV-C LED array, taken using a silicon photodiode, with only the top UV-LEDs turned on, and keeping the testing conditions the same as the microbial tests. Figure 14 shows the measured data obtained at four different working distances (WDs).

The minimum irradiance was measured to be 2 mW/cm^2^ with only the top UV-LEDs turned on, assuming a worst-case scenario. The minimum to maximum irradiance ratio was 0.8, implying good uniformity throughout the measurement area.

#### 4.3.2 Microbial Validation

**Fig. 15:**
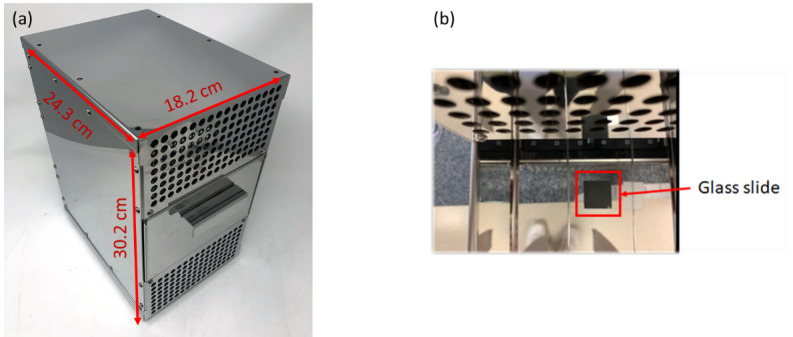
(a) Photograph of the prototype device and (b) glass slide placed inside the tray.

Microbial validation was performed against the MS2 bacteriophage. The MS2 bacteriophage was chosen because it is considered to be a surrogate for influenza viruses. It is a hardy virus and exhibits high UV resistance, representing the worst-case scenario for UV efficacy testing [36]. This bacteriophage is quick to propagate and is easier to handle in a level 2 safety laboratory. The prototype device was tested against a modified version of the ASTM E3135-18 “Standard Practice for Determining Antimicrobial Efficacy of Ultraviolet Germicidal Irradiation Against Microorganisms on Carriers with Simulated Soil” test method [37], but no soiling conditions were tested. The irradiance measurements were performed in-house earlier and not as a part of this third-party laboratory test. The carriers used for this testing were 10 mm × 10 mm painted glass slides, which were used as a surrogate for nonporous opaque surfaces. The photograph of the prototype device is shown in Fig. 15(a), while the placement of the glass slide inside the tray is shown in Fig. 15(b).

The antimicrobial activity (*R* value) of the test agent to the microbe was calculated according to Eq. (3).

*R = A_0_* − *A_t_* (3)

where *R* is the value of antimicrobial activity, or log reduction of the test agent;

*A_0_* is the logarithm of the number of viable bacteria, in PFU/swatch (or PFU/coupon), where PFU is plaque-forming unit, initially (*t*=0) recovered from the phosphate-buffered water control; and

*A_t_* is the logarithm of the number of viable bacteria, in PFU/swatch (or PFU/coupon), recovered from the treated test agent after the specified contact time.

Table 3 shows the preliminary testing results obtained at 23 s and at 2 min cycle time with the prototype device.

**Table 3:** Glass slide (carrier) test results where only the top array of UV-LEDs is illuminated.

**Test Parameter**	**Control** **(No LED)**	**Exposure Time**	
		23 s	2 min
**Carrier 1 (PFU/carrier)^a^**	445,000	1030	3560^b^
**Carrier 2 (PFU/carrier)**	880,000	320	50
**Carrier 3 (PFU/carrier)**	1,005,000	8800	170
**Log Rep 1**	5.65	3.01	3.55^b^
**Log Rep 2**	5.94	2.51	1.70
**Log Rep 3**	6.00	3.94	2.23
**Average**	5.87	3.15	2.49
***A*_0_**	5.87		
***A*_t_**		3.15	2.49
***R***		2.71	3.37
**% reduction**		99.8054%	99.9575%

^a^ PFU is plaque-forming unit.

^b^ We are not able to explain the observed increase in values for carrier 1.

While these preliminary microbial testing results indicate that a 2.71 log_10_ reduction (99.8%) could be obtained with a UV dose of 58 mJ/cm^2^ and a 3.37 log_10_ reduction (99.96%) could be obtained with a UV dose of 300 mJ/cm^2^ using the prototype device, we acknowledge that further testing with a greater number of replicates is needed to further prove the effectiveness of the device with greater confidence. Testing with other HAI-causing microbes is also intended for a future study.

## 5 Conclusions

**Fig. 16:**
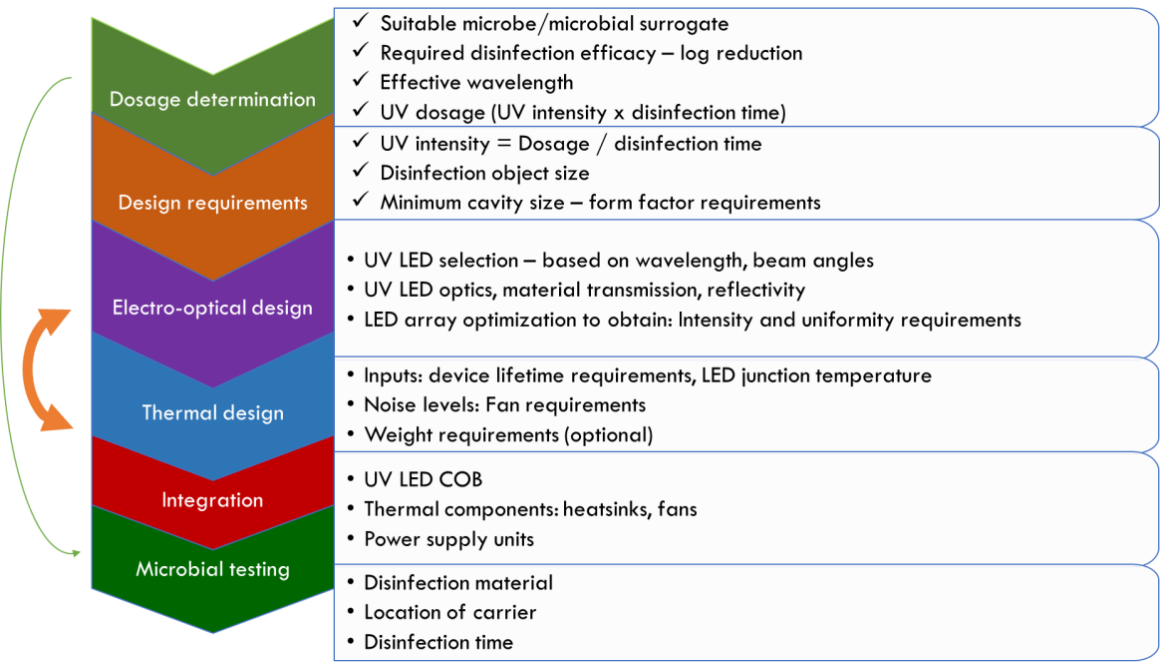
Summary of the overall design process for a surface disinfection device.

While the radiant efficiency of UV-C LEDs is lower than that of mercury lamps, rapid progress in the LED technology demonstrates tremendous potential for development of disinfection products. Commercially available UV-C LEDs are suitable to be used for developing surface disinfection devices. However, the electro-optical, mechanical, and thermal components must be integrated and designed in tandem to achieve optimal performance and therefore effective disinfection. This study showed that product designers need to thoroughly understand UV-LED datasheet parameters for use in product development. The overall development process is summarized in Fig. 16.

It is essential to highlight the importance of thermal management to obtain target lifetimes and technologies such as the thermal pad technology, which can aid drastically in achieving lower junction temperatures. Device design is nontrivial and requires multidisciplinary knowledge as well as a thorough determination of user targets to develop an effective product. In addition, validation methods need to be identified based on standard and approved practices.

## References

[1] Bolton JR, Cotton CA (2008) The Ultraviolet Disinfection Handbook (American Water Works Association, Denver CO). 1st Ed (2nd Ed 2011).

[2] Magill SS, O’Leary E, Janell SJ, Thompson DL, Dumyati G, Nadle J, Wilson LE, Kainer MA, Lynfield R, Greissman S, Ray SM, Beldavs Z, Gross C, Bamberg W, Sievers M, Concannon C, Buhr N, Warnke L, Maloney M, Ocampo V, Brooks J, Oyewumi T, Sharmin S, Richards K, Rainbow J, Samper M, Hancock EB, Leaptrot D, Scalise E, Badrun F, Phelps R, Edwards JR, Emerging Infections Program Hospital Prevalence Survey Team (2018) Changes in prevalence of health care–associated infections in U.S. hospitals. The New England Journal of Medicine 379(18):1732–1744. 30380384 10.1056/NEJMoa1801550 PMC7978499

[3] Poster DL, Miller CC, Martinello RA, Horn NR, Postek MT, Cowan TE, Obeng YS, Kasianowicz JJ (2021) Ultraviolet radiation technologies and healthcare-associated infections: Standards and metrology needs. Journal of Research of the National Institute of Standards and Technology 126:126014. 10.6028/jres.126.014 PMC10046890 38469449

[4] Boone SA, Gerba CP (2007) Significance of fomites in the spread of respiratory and enteric viral disease. Applied and Environmental Microbiology 73(6):1687–1696. 17220247 10.1128/AEM.02051-06 PMC1828811

[5] Stephens B, Azimi P, Thoemmes MS, Heidarinejad M, Allen JG, Gilbert JA (2019) Microbial exchange via fomites and implications for human health. Current Pollution Reports 5:198–213. 34171005 10.1007/s40726-019-00123-6 PMC7149182

[6] Centers for Disease Control and Prevention (CDC) (2019) *CDC Study: Antibiotic Resistance Threats in the United States* (CDC, Atlanta, GA). Available at https://www.cdc.gov/drugresistance/pdf/threats-report/2019-ar-threats-report-508.pdf

[7] Dewey HM, Jones JM, Keating MR, Budhathoki-Uprety J (2022) Increased use of disinfectants during the COVID-19 pandemic and its potential impacts on health and safety. ACS Chemical Health & Safety 29(1):27–38.

[8] Coohill TP, Sagripanti JL (2008) Overview of the inactivation by 254 nm ultraviolet radiation of bacteria with particular relevance to biodefense. Photochemistry and Photobiology 84(5):1084–1090. 18627518 10.1111/j.1751-1097.2008.00387.x

[9] Rice KM, Walker EM Jr, Wu M, Gillette C, Blough ER (2014) Environmental mercury and its toxic effects. Journal of Preventive Medicine and Public Health 47(2):74–83. 24744824 10.3961/jpmph.2014.47.2.74 PMC3988285

[10] Muramoto Y, Kimura M, Nouda S (2014) Development and future of ultraviolet light-emitting diodes: UV-LED will replace the UV lamp. Semiconductor Science and Technology 29:084004. Available at https://iopscience.iop.org/article/10.1088/0268-1242/29/8/084004

[11] Kreitenberg A, Martinello RA (2021) Perspectives and recommendations regarding standards for ultraviolet-C whole-room disinfection in healthcare. Journal of Research of the National Institute of Standards and Technology 126:126015. 10.6028/jres.126.015 PMC9681192 36475087

[12] Kowalski W (2009) Ultraviolet Germicidal Irradiation Handbook (Springer, Berlin).

[13] International Commission on Illumination (CIE) (2003) CIE 155:2003—Ultraviolet Air Disinfection (CIE Central Bureau, Vienna, Austria).

[14] Bolton J (2017) Action spectra—A review. *IUVA News* 19(2):10–12 and supplementary file. Available at https://uvsolutionsmag.com/stories/pdf/archives/190201-IUVA_News_Summer2017_final_ActionSpectra_combo.pdf

[15] Beck SE (2015) Wavelength-Specific Effects of Ultraviolet Light on Microorganisms and Viruses for Improving Water Disinfection. Ph.D. Thesis. (University of Colorado, Boulder, CO). Available at https://scholar.colorado.edu/downloads/rn301175t

[16] Biasin M, Bianco A, Pareschi G, Cavalleri A, Cavatorta C, Fenizia C, Galli P, Lessio L, Lualdi M, Tombetti E, Ambrosi A, Redaelli EMA, Saulle I, Trabattoni D, Zanutta A, Clerici M (2021) UV-C irradiation is highly effective in inactivating SARS-CoV-2 replication. Scientific Reports 11:6260. UV-LED 33737536 10.1038/s41598-021-85425-w PMC7973506

[17] Blatchley ER, Petri B, Sun W (2021) SARS-CoV-2 ultraviolet radiation dose-response behavior. Journal of Research of the National Institute of Standards and Technology 126:126018. 10.6028/jres.126.018 PMC10857211 38469447

[18] Templeton MR, Antonakaki M, Rogers M (2009) UV dose–response of *Acinetobacter baumannii* in water. *Environmental Engineering Scienc*e 26(3):697–701.

[19] Masjoudi M, Mohseni M, Bolton JR (2021) Sensitivity of bacteria, protozoa, viruses, and other microorganisms to ultraviolet radiation. Journal of Research of the National Institute of Standards and Technology 126:126021. 10.6028/jres.126.021 PMC11259122 39081635

[20] Skowron K, Bauza-Kaszewska J, Dobrzański Z, Paluszak Z, Skowron KJ (2014) UV-C radiation as a factor reducing microbiological contamination of fish meal. The Scientific World Journal 2014:928094. 10.1155/2014/928094 PMC3918690 24578670

[21] Geldert A, Balch HB, Gopal A, Su A, Grist SM, Herr AE (2021) Best practices for germicidal ultraviolet-C dose measurement for N95 respirator decontamination. Journal of Research of the National Institute of Standards and Technology 126:126020. 10.6028/jres.126.020 PMC10046750 38469452

[22] Chandran ML, Ramamurthy PC, Kanjo K, Narayan R, Menon SR (2021) Efficacy of ultraviolet-C devices for the disinfection of personal protective equipment fabrics and N95 respirators. Journal of Research of the National Institute of Standards and Technology 126:126023. 10.6028/jres.126.023 PMC9681524 36475082

[23] Violumas (2021) *UV-LED Datasheet Sample* (Violumas, Fremont, CA). Available at https://www.violumas.com/wp-content/uploads/2021/08/VS5252C48L3-265-Rev080621.pdf

[24] Lombini M, Diolaiti E, De Rosa A, Lessio L, Pareschi G, Bianco A, Cortecchia F, Fiorini M, Fiorini G, Malaguti G, Zanutta A (2021) Design of optical cavity for air sanification through ultraviolet germicidal irradiation. Optics Express 29(12):18688–18704. 34154120 10.1364/OE.422437

[25] Stojalowski PS, Fairfoull J (2021) Comparison of reflective properties of materials exposed to ultraviolet-C radiation. Journal of Research of the National Institute of Standards and Technology 126:126017. 10.6028/jres.126.017 PMC10856222 38469450

[26] Yates SF, Isella G, Rahislic E, Barbour S, Tiznado L (2021) Effects of ultraviolet-C radiation exposure on aircraft cabin materials. Journal of Research of the National Institute of Standards and Technology 126:126019. 10.6028/jres.126.019 PMC10855778 38469438

[27] Wang Y, Alonso JM, Ruan X (2017) A review of LED drivers and related technologies. IEEE Transactions on Industrial Electronics 64(7):5754–5765.

[28] Lasance CJM, Poppe A (2014) Thermal Management for LED Applications (Springer, New York, NY).

[29] Cree LED (2021) Cree XLamp LEDs (Cree LED, Durham, NC). Available at https://cree-led.com/media/documents/XLamp_PCB_Thermal.pdf

[30] Quadica (2021) Luxeon Star LEDs (Quadica, Lethbridge, Alberta, Canada). Available at https://www.luxeonstar.com/sinkpad

[31] Luo X, Liu S (2007) A microjet array cooling system for thermal management of high-brightness LEDs. IEEE Transactions on Advanced Packaging 30(3):475–484.

[32] Hamida MBB, Hatami M (2021) Optimization of fins arrangements for the square light emitting diode (LED) cooling through nanofluid-filled microchannel. Scientific Reports 11:12610. 34131229 10.1038/s41598-021-91945-2 PMC8206350

[33] Fredes P, Raff U, Gramsch E (2020) Intelligent heat extraction with thermoelectric cooler for UV-C LEDs. *UV Solutions* Quarter 2. Available at https://uvsolutionsmag.com/articles/2020/intelligent-heat-extraction-with-thermoelectric-cooler-for-uv-c-leds/

[34] Cadnum J, Pearlmutter B, Redmond S, Jencson A, Benner K, Donskey C (2021) Ultraviolet-C (UV-C) monitoring made simple: Colorimetric indicators to assess delivery of UV-C light by room decontamination devices. Infection Control & Hospital Epidemiology 1-6. 10.1017/ice.2021.113 33858538

[35] Stojalowski PS, Jonathan F (2021) Comparison of reflective properties of materials exposed to ultraviolet-C radiation. Journal of Research of the National Institute of Standards and Technology 126:126017. 10.6028/jres.126.017 PMC10856222 38469450

[36] U.S. Environmental Protection Agency (2020) *Results for SARS-CoV-2 Surface Disinfection with UV-November 10, 2020* (U.S. EPA, Washington, D.C.). Available at https://www.epa.gov/covid19-research/results-sars-cov-2-surface-disinfection-uv-november-10-2020

[37] ASTM International (2018) *ASTM E3135-18—Standard Practice for Determining Antimicrobial Efficacy of Ultraviolet Germicidal Irradiation Against Microorganisms on Carriers with Simulated Soil* (ASTM International, West Conshohocken, PA). Available at https://www.astm.org/Standards/E3135.htm

